# GATA3 Predicts the Tumor Microenvironment Phenotypes and Molecular Subtypes for Bladder Carcinoma

**DOI:** 10.3389/fsurg.2022.860663

**Published:** 2022-05-12

**Authors:** Qixin Zhang, Tiezheng Qi, Yu Long, Xiaowen Li, Yiyan Yao, Qi Wu, Anrong Zou, Belaydi Qthmane, Peihua Liu

**Affiliations:** ^1^Department of Urology, Ping Kuang General Hospital, Pingxiang, China; ^2^Xiangya School of Medicine, Central South University, Changsha, China; ^3^Department of Urology, Xiangya Hospital, Central South University, Changsha, China; ^4^National Clinical Research Center for Geriatric Disorders, Xiangya Hospital, Changsha, China

**Keywords:** GATA3, bladder cancer, tumor microenvironment, immunotherapy, risk score, chemotherapy

## Abstract

**Aims:**

GATA3 is a key player in antitumor immunology, and continuous studies show that it might be a key biomarker for bladder cancer (BLCA). Thus, we lucubrate the immunological role of GATA3 in BLCA.

**Main Methods:**

We initially used pan-cancer analysis to analyze the expression pattern and immunological function of GATA3 with data gathered from the TCGA (The Cancer Genome Atlas). Then, in the BLCA tumor microenvironment (TME), we comprehensively associated GATA3 with immunomodulators, cancer immune cycles, tumor-infiltrating immune cells (TIICs), immune checkpoints, and T-cell inflamed scores(TIS). The role of GATA3 in predicting BLCA molecular subtypes and responsiveness to various treatment regimens was also investigated. We confirmed our findings in an external cohort and the Xiangya-Pingkuang cohort to guarantee the correctness of our study.

**Key Findings:**

GATA3 was preferentially expressed in the TME of numerous malignancies, including BLCA. High GATA3 expression was adversely connected with immunological aspects such as immunomodulators, cancer immune cycles, TIICs, immune checkpoints, and TIS in the BLCA TME. In addition, high GATA3 was more likely to be a luminal subtype, which meant it was less susceptible to cancer immunotherapy and neoadjuvant chemotherapy but more sensitive to targeted treatments.

**Significance:**

GATA3 may aid in the precision treatment for BLCA because it can accurately predict the clinical outcomes and the TME characteristics of BLCA.

## Introduction

Bladder cancer (BLCA) is a common urinary malignancy with significant heterogeneity. There are nearly 550,000 new cases and 200,000 new deaths each year (1). Notwithstanding various treatment options for BLCA, such as surgery, chemotherapy, immunotherapy, targeted therapy, and radiotherapy, the overall survival of advanced BLCA is still discouraging because of the primary or required treatment resistance (2). Hence, exploring new treatment response indicators and prognostic biomarkers for BLCA is imperative for urologists to achieve individual precision medicine.

GATA transcription factor family consists of six members, in which GATA3 plays a vital regulatory role in the initiation and progression of multiple cancers (3). GATA3, known as a specific marker of bladder urothelial carcinoma, is significantly highly expressed in BLCA (4, 5). Although GATA3 is highly expressed in bladder cancer, its high expression may adversely inhibit the progression of tumor cells in BLCA (6). Meanwhile, the over-expression of GATA3 predicts a lower tumor stage and a lower tumor grade but a higher relapse-free survival rate (7, 8). Such an imbalance between the over-expression profile of GATA3 in BLCA cancer tissues and the inhibitory function of GATA3 on bladder cancer cells may be explained by the ability of GATA3 to transform basal bladder cancer cells into luminal cells (7, 9, 10). Universally, the basal bladder cancer subtypes had a poorer prognosis compared with the luminal bladder cancer subtypes (11, 12).

Nowadays, GATA3 has been reported to play a critical role in regulating the anti-cancer immune response in the tumor microenvironment (TME) (13–15). However, the associations between GATA3 and the tumor immune phenotypes in BLCA remain to be further elucidated.

## Materials and Methods

Supplementary Figure 1A shows the workflow of this study.

### Data Sets Collection and Preprocessing

#### Training Cohort

The TCGA-BLCA mRNA expression data (FPKM) value and corresponding clinicopathologic information were downloaded from The Cancer Genome Atlas (TCGA) (https://portal.gdc.cancer.gov/). The FPKM value was translated into transcripts per kilobase million (TPM) value. Finally, we gathered 410 BLCA samples and 19 normal tissues in total.

#### The Xiangya-Pingkuang Cohort

The Xiangya-Pingkuang cohort was collected from our hospital. This data set has been uploaded to the GEO database (GSE188715). A total of 57 BLCA cancer samples and 13 normal bladder samples were collected in this cohort. We used the TPM value of the cohort for external validation.

#### IMvigor210 Cohort

IMvigor210 cohort was a BLCA immunotherapy-related cohort. The mRNA expression data and corresponding clinical information were obtained from http://research-pub.Gene.com/imvigor210corebiologies/ based on the Creative Commons 3.0 License.

### The Expression and Prognosis Profiles of GATA3 in Pan-Cancers

We first analyzed the pan-cancer expression profiles of GATA3 expression between cancer and normal tissues from the TCGA database. Then, we performed survival analyses based on the GATA3 expression and calculated the HR (hazard ratios) with *P*-value for OS (overall survival). We studied results from pan-cancer analysis to confirm the candidate target cancer with differential expression of GATA3, placing a special focus on BLCA. We subsequently correlated GATA3 with several clinicopathologic characteristics, including grade, stage, sex, and histologic subtypes, in both the TCGA-BLCA and Xiangya-Pingkuang cohorts.

### Evaluation of the Immunological Role of GATA3 in Pan-Cancers

We evaluated the correlations between GATA3 and 122 immunomodulators in 33 cancers from the TCGA database. Subsequently, correlations were also analyzed between GATA3 and four critical immune checkpoints, including PD-1, PD-L1, CTLA-4, and LAG-3 in pan-cancers. Finally, we further correlated GATA3 with 28 cancer-associated immune cells estimated using the ssGSEA algorithm.

### Immunological Role of GATA3 in BLCA

After performing pan-cancer analyses, we focused on the BLCA in which GATA3 had the strongest negative correlations with tumor immune status. Similarly, we compared the differences in the expression of 122 immunomodulators between low-GATA3 and high-GATA3 groups. Afterward, we analyzed the distribution of the anti-cancer immune response activities between low and high GATA3 groups; the vigor of the anti-cancer immune response steps determined the fate of tumor cells. A total of seven critical steps were analyzed in this study (16–18). Besides, we correlated GATA3 with tumor-infiltrating lymphocytes (TILs) and corresponding effector genes. In addition, we correlated GATA3 with immune checkpoint inhibitors (ICIs), including PD-1, PD-L1, CTLA-4, and LAG-3. Finally, we also associated GATA3 with the T-cell inflamed score, which represents the level of pre-existing cancer immunity (16, 19). These immune characters were described in detail in our previous studies (16, 17).

### Depicting the Molecular Subtypes of BLCA and Collecting Several Therapeutic Response Signatures

Seven molecular subtypes systems of BLCA, including UNC, Baylor, TCGA, MDA, Lund, CIT, and Consensus subtype systems, were well developed (11, 12, 20–24). We determined the subtype of an individual sample with two R packages, namely ConsensusMIBC and BLCA subtyping (12). Thenceforth, correlations between GATA3 with different molecular subtypes and subtypes specific signatures were explored. Receiver operating characteristic(ROC) curves were plotted for evaluating the accuracy of GATA3 in predicting BLCA molecular subtypes. In addition, we included several therapeutic response related signatures or molecules, such as the mutational profiles related to neoadjuvant chemotherapy response and the gene signatures associated with the response of targeted therapies and radiotherapy. Finally, we collected many drug targets from the Drug Bank database. These therapeutic response related signatures or molecules were reported in our previous studies (16, 17).

### Statistical Analysis

The data visualization and statistical analyses were performed in R software (Version: 4.0.5). Analyses with two-sided *P* <0.05 were considered statistically significant. Correlations between variables were explored using Pearson or Spearman coefficients. We used *t*-test to compare continuous variables fitting a normal distribution between binary groups, and the chi-squared test or Fisher's exact test to compare categorical variables. The Kaplan-Meier method was applied to plot the survival curves, and the log-rank test was used to calculate statistical significance. We calculated the accuracy of the risk score in predicting survival and molecular subtypes by plotting the ROC curves.

## Results

### Pan-Cancer Expression Profiles and Prognostic Significance of GATA3

**Figure 1:**
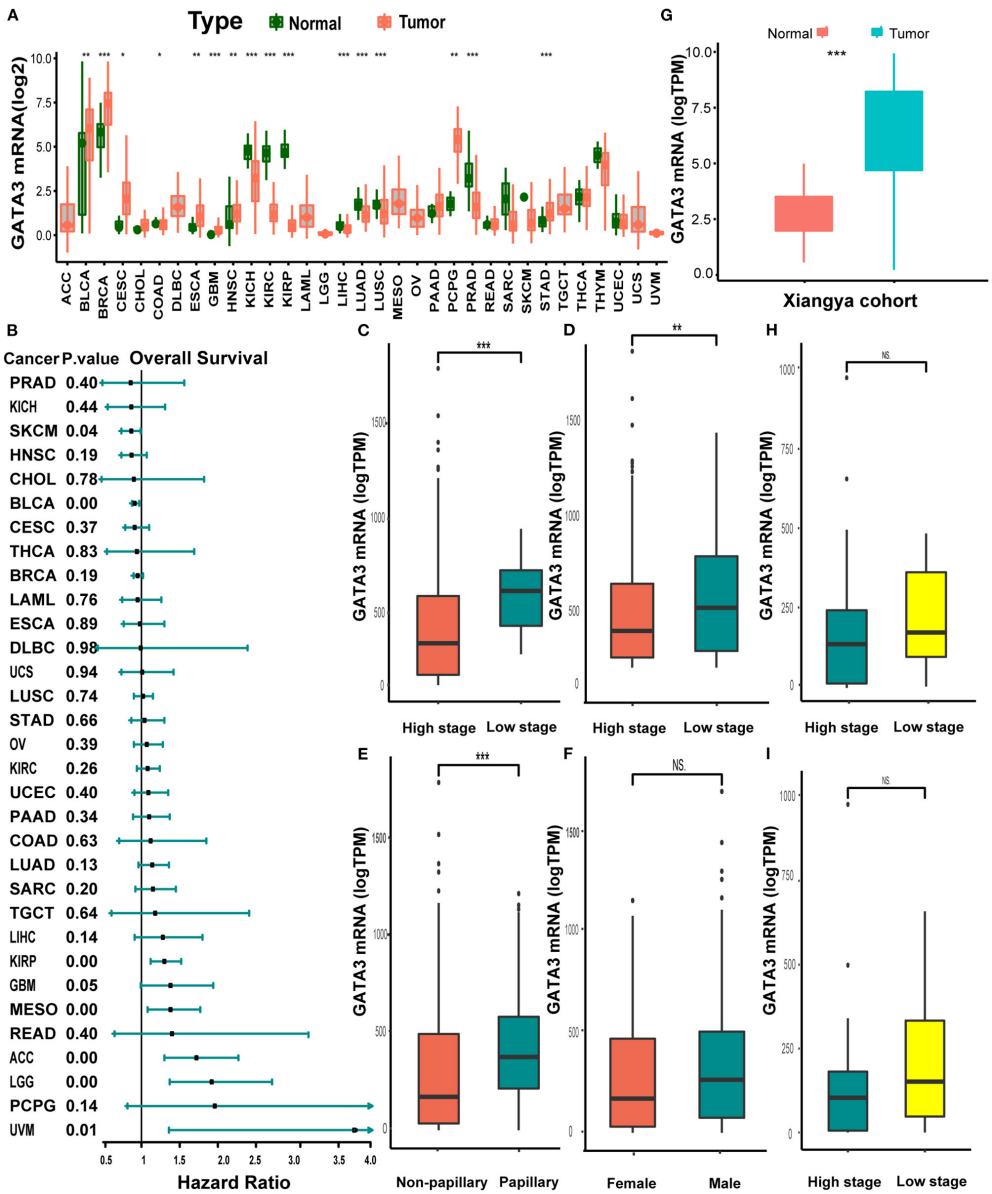
Pan-cancer expression profiles and prognostic significance of GATA3. **(A)** GATA3 expression between normal tissues and tumor tissues in 33 various cancers. **(B)** Hazard ratios of overall survival analysis in various cancers with univariate cox analysis. **(C–F)** Clinicopathological correlation analysis of GATA3 in the TCGA-BLCA cohort. (The analysis was done using the *T*-test. * *P* <0.05, ** represented a *P* <0.01, *** represented a *P* <0.001, and NS signified a P > 0.05). **(G)** Expression analysis of GATA3 in the Xiangya-Pingkuang cohort. **(H,I)**. Correlation analysis of clinicopathological characteristics of GATA3 in the Xiangya-Pingkuang cohort.

**Figure 2:**
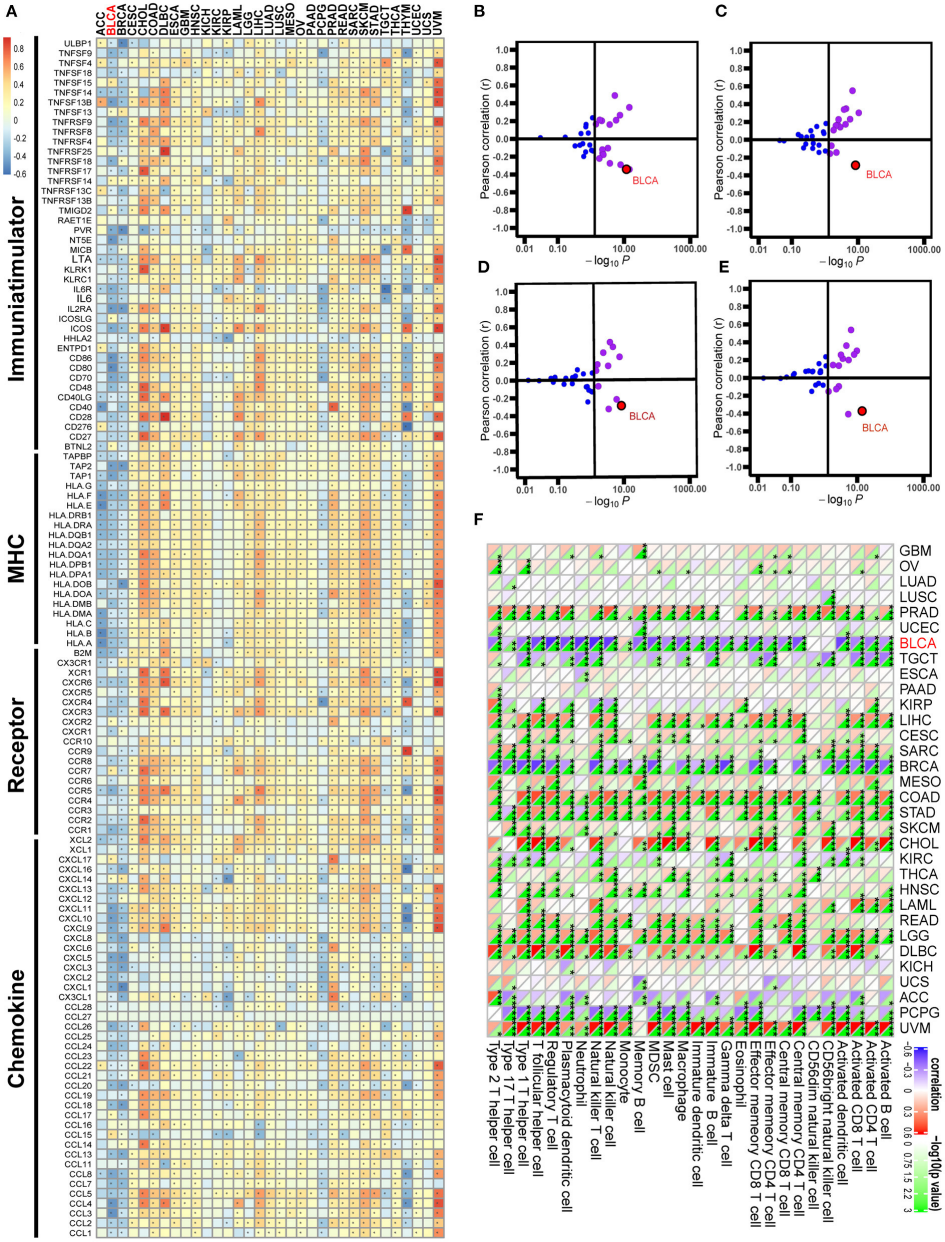
Correlations between GATA3 with immunological characteristics in pan-cancers. **(A)** Correlation between GATA3 and 122 immunomodulators (chemokines, receptors, MHC, and immunostimulators). **(B–E)** Correlation between GATA3 and four immune checkpoints, namely PD-L1, CTLA-4, PD-1, and LAG-3. **(F)** Correlation between Siglec15 and 28 tumor-associated immune cells calculated with the ssGSEA algorithm.

Pan-cancer analysis of data from TCGA revealed that GATA3 was overexpressed in tumor tissues than in normal tissues within several carcinomas, including BLCA, breast cancer, and others (Figure 1A). In contrast, GATA3 was down-expressed in renal cancers, prostate carcinoma, and others (Figure 1A). Therefore, colossal heterogeneity existed in the expression profiles of GATA3 in pan-cancers. Similar huge heterogeneity existed on the prognostic correlations of GATA3 in pan-cancers (Figure 2B); especially for BLCA, as the hazard ratio was <1 with a *p*-value <0.01 (Figure 1B). Single-cell analysis further verified that GATA3 was more highly-expressed in tumor tissues in comparison to normal tissues (Supplementary Figure 1B). Consistently, in BLCA, correlation analyses based on the TCGA-BLCA cohort between GATA3 and several clinicopathologic characteristics indicated statistical significance. GATA3 was highly expressed in patients with low-grade and low tumor stages (Figures 1C,D). The expression level was obviously high in papillary BLCA tissues compared to non-papillary tissues (Figure 1E). Meanwhile, the expression of GATA3 in the majority of the male patients was more elevated than in females (Figure 1F). Further validations based on the Xiangya-Pingkuang cohort verified that GATA3 was highly expressed in BLCA (Figure 1G). As for clinicopathologic features, a higher expression level of GATA3 in patients with a low grade and low stage was as expected as well, though the difference was not statistically significant (Figures 1H,I).

### Pan-Cancer Immunological Correlation Analysis of GATA3

Pan-cancer analysis contributed to illustrating the immunological role of GATA3 and screening the types of cancers that may benefit from anti-GATA3 immunotherapy. The correlations between GATA3 and the immunomodulators were heterogeneous in different cancers (Figure 2A). Overall, GATA3 was positively correlated with the immune status of most cancers, such as uveal melanoma, colon adenocarcinoma, and cholangiocarcinoma. But in BLCA, GATA3 was negatively correlated with most immunomodulators, including immunomostimulators (such as TNF, ULBP1, and CD276), MHC (such as HLA), receptors, and chemokines (Figure 2A). Besides, our results demonstrated that the expression of GATA3 was mutually exclusive of several critical immune checkpoints, including PD-L1, PD-1, CTLA-4, and LAG-3 in BLCA (Figures 2B–E). Furthermore, we evaluated the infiltration level of TIICs in TME of various cancers with the ssGSEA algorithm; results indicated significant negative correlations between GATA3 and most of TIICs, such as activated CD8 T-cells and activated DC cells in BLCA (Figure 2F). As was known to all, there are many similar biological behaviors between bladder cancer and breast cancer (23, 25, 26). Consistently, we found that GATA3 was also negatively related to the immunological characters of breast cancer.

### GATA3 Shapes a Cold TME in BLCA

**Figure 3:**
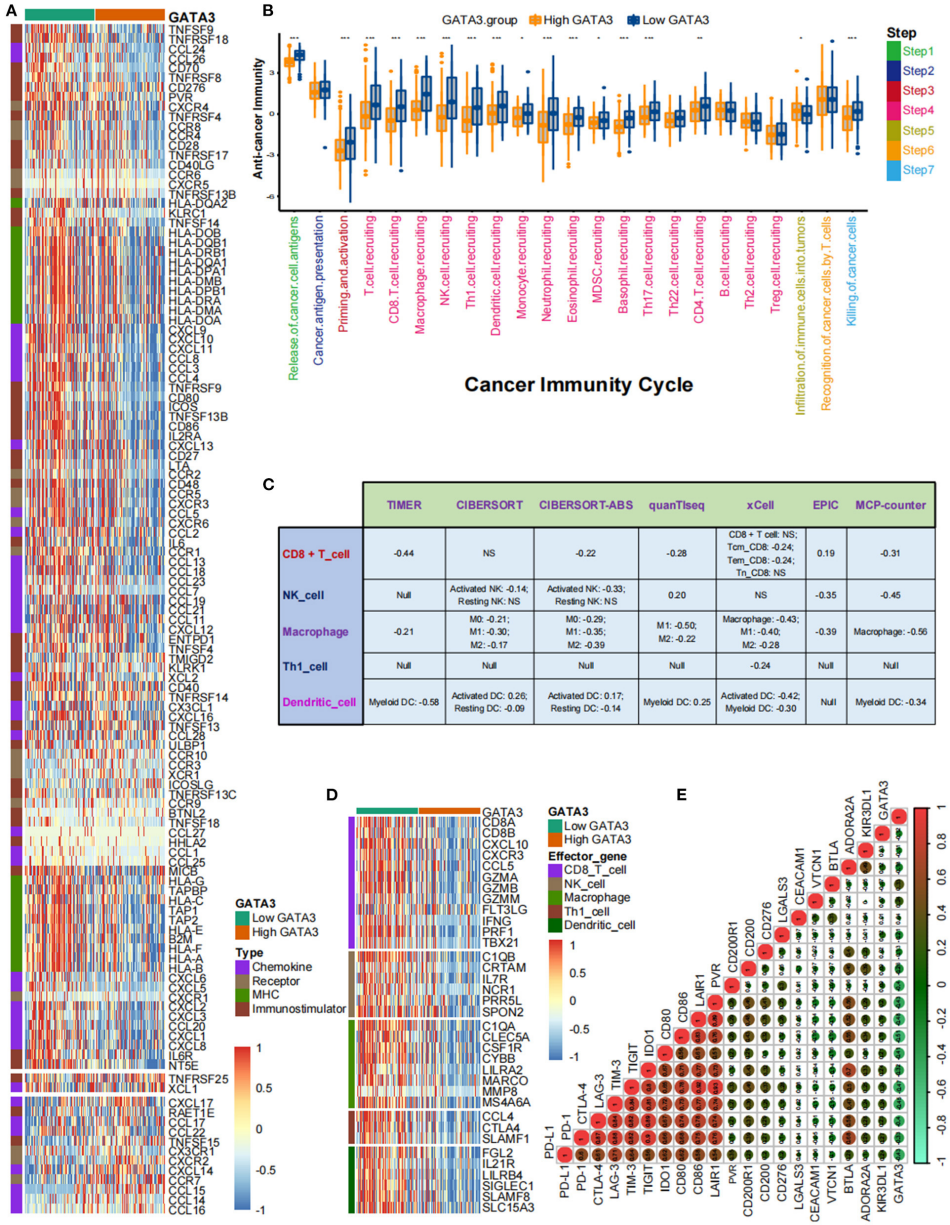
GATA3 correlated with the tumor immune microenvironment in BLCA. **(A)** Differential expression of GATA3 between high- and low-GATA3 tissues in BLCA. **(B)** Differential expression of GATA3 among various steps of the anti-tumor immune cycle. **(C)** Correlation between GATA3 and the infiltration levels of five TIICs with various algorithms. **(D)** Differential expression of effector genes of five mentioned TIICs between high- and low-GATA3 tissues in BLCA. **(E)** Correlation between GATA3 and 20 inhibitory immune checkpoints.

Pan-cancer analyses screened that GATA3 was negatively correlated with the immune status of several cancers, such as breast cancer and BLCA. In this section, we ulteriorly revealed that most immunomodulators were down-regulated in high-GATA3 tissues compared to low-GATA3 tissues (Figure 3A). A significant number of MHC molecules were lowly expressed in high-GATA3 tissues, indicating a decline in the capacity to present and process tumor antigens in the high-GATA3 group. CXCL9, CXCL10, CXCL11, and CXCR3 recruited and activated immune cells, such as CD8 + T-cells, to suppress tumor progression through CXCL9,-10−11/CXCR3 axis, and they were down-regulated in the high-GATA3 group (27). Also, Chemokines including CCL2, CCL3, and their paired receptors, including CCR2 and CCR3, were conducive to promoting the recruitment of TIICs, such as antigen-presenting cells. And these chemokines and corresponding receptors were down-regulated in the high-GATA3 group. However, CXCL8, which promoted tumor invasion and angiogenesis (28), was not significantly down-regulated in the high-GATA3 group. Confirmation of the immunological role of GATA3 in TME based on the relationship between GATA3 and individual chemokines or receptors was actual overgeneralization because of the complicacy and comprehensiveness of the chemokine system.

**Figure 4:**
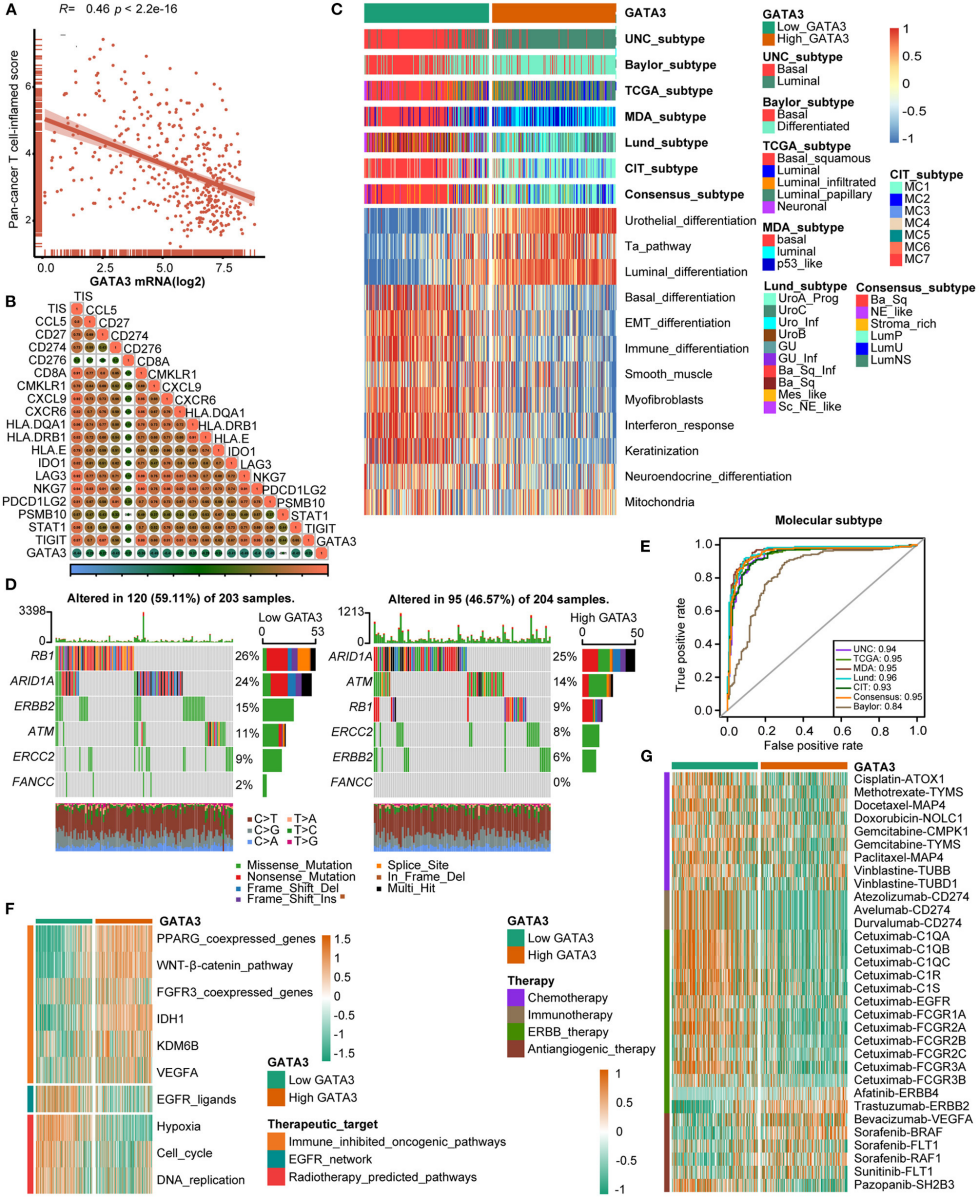
GATA3 predicts the molecular subtype and response to several therapies in BLCA. **(A,B)** Correlation between GATA3 with TIS and the corresponding TIS-related effector genes. **(C)** Correlations between GATA3 and molecular subtypes with seven different subtyping systems. **(D,E)** Mutational profiles of neoadjuvant chemotherapy-related genes in low- and high-GATA3 tissues. **(E)** ROC analysis on the prediction accuracy of GATA3 for molecular subtypes with different systems. **(F)** Correlation between GATA3 and the enrichment scores of therapeutic signatures. **(G)** Correlation between GATA3 and the drug-target genes of various therapies.

The cancer immunity cycle was a comprehensive system integrating the roles of various chemokines and other immunomodulators (18). We noticed that activities of most steps, including cancer antigen presentation, T cell priming, and immune cell recruitment, were significantly restrained in the high-GATA3 group (Figure 3B). Subsequently, the correlation between the expression of GATA3 and TILs proved that high GATA3 indicated a low immune infiltration level (Figure 3C). We also consistently explored the correlation between GATA3 and effector genes of TILs. Pervasive down-regulation of genes was shown in the high-GATA3 group (Figure 3D). Likewise, GATA3 was negatively correlated with most immune checkpoint inhibitors, including PD-L1, PD1, CTLA4, LAG-3, and TIGHT (Figure 3E). In addition, we demonstrated that GATA3 was negatively correlated with the pan-cancer T-cell inflamed score (Spearman *R* = −0.46) (Figure 4A). Furthermore, we discovered negative relationships between GATA3 and all individual genes included in the T cell inflamed signature (Figure 4B). These results revealed that high GATA3 indicated a cold TME in BLCA.

### GATA3 Predicts Molecular Subtypes and Therapeutic Opportunities

Subtype classification in BLCA showed that the basal subtype was more likely to respond to ICB (immune checkpoint blockade) therapy because of higher immune infiltration than the luminal subtype (12, 29, 30). Unambiguous identification of the tumor subtypes in BLCA aids in precision clinical therapy. We explored the associations between the tumor subtypes and GATA3 expression (Figure 4C), and our results revealed that BLCA with high GATA3 expression was more inclined to be luminal subtype. Inversely, tumor tissues with low GATA3 expression were positively correlated with the basal subtype while indicating a high immune infiltration level. As shown in Figure 4C, enrichment scores for urothelial differentiation, Ta pathway, and luminal differentiation were superior in the high-GATA3 group. In contrast, enrichment scores for basal differentiation, EMT (epithelial-mesenchymal transition) differentiation, and immune differentiation were lower in the high-GATA3 group; a luminal subtype of BLCA with high GATA3 predicted a weaker response to ICB therapy. Additionally, the AUCs of GATA3 in predicting molecular subtypes of six independent systems were ≥0.90 (Figure 4E). We ulteriorly perform molecular subtype analysis in GSE31684 and GSE32894 to ensure the correctness of the results (Supplementary Figures 1C–F).

The capacity of molecular subtypes to predict neoadjuvant chemotherapy, radiotherapy, and several targeted therapies was illustrated clearly (12, 29, 31). Gene mutation was a typical characteristic of tumor heterogeneity. Foundamentally, we focused on comparing the differences in mutation frequencies of genes associated with NAC efficacy between the high and low GATA3 groups. In our study, we found high mutation rates of RB1 (retinoblastoma protein 1), ARID1A (AT-rich interacting domain-containing protein 1A), and ERBB2 (erythroblastic leukemia viral oncogene homolog 2) in the low GATA3 group, while the high mutation rates of ARID1A, ATM, and RB1 in the high GATA3 group (Figure 4D). Notably, ARID1A and RB1 showed high mutation rates in both patient groups. These results suggested that the mutation frequency of RB1 in the high- GATA3 group was significantly lower than that in the low GATA3 group. In contrast, no difference occured in the mutation frequency of ARIDA1A. Tumors with high GATA3 tumors may be more insensitive to NAC. Besides, enrichment scores for radiotherapy-predicted pathways and EGFR ligands were higher in the low-GATA3 group. By contrast, enrichment scores for immune inhibited oncogenic pathways were greater in the high-GATA3 group (Figure 4F). Furthermore, an analysis performed from the Drugbank database revealed a significantly higher response to chemotherapy, immunotherapy, and ERBB therapy in the low-GATA3 group, while a lower response to antiangiogenic therapy (Figure 4G). Results indicated that ICB, neoadjuvant or adjuvant chemotherapy, and ERBB therapy were positive treatment options for patients with low GATA3 expression.

### Reverification of the Role of GATA3 in Predicting Immune Phenotypes and BLCA Molecular Subtypes in the Xiangya-Pingkuang Cohort

**Figure 5:**
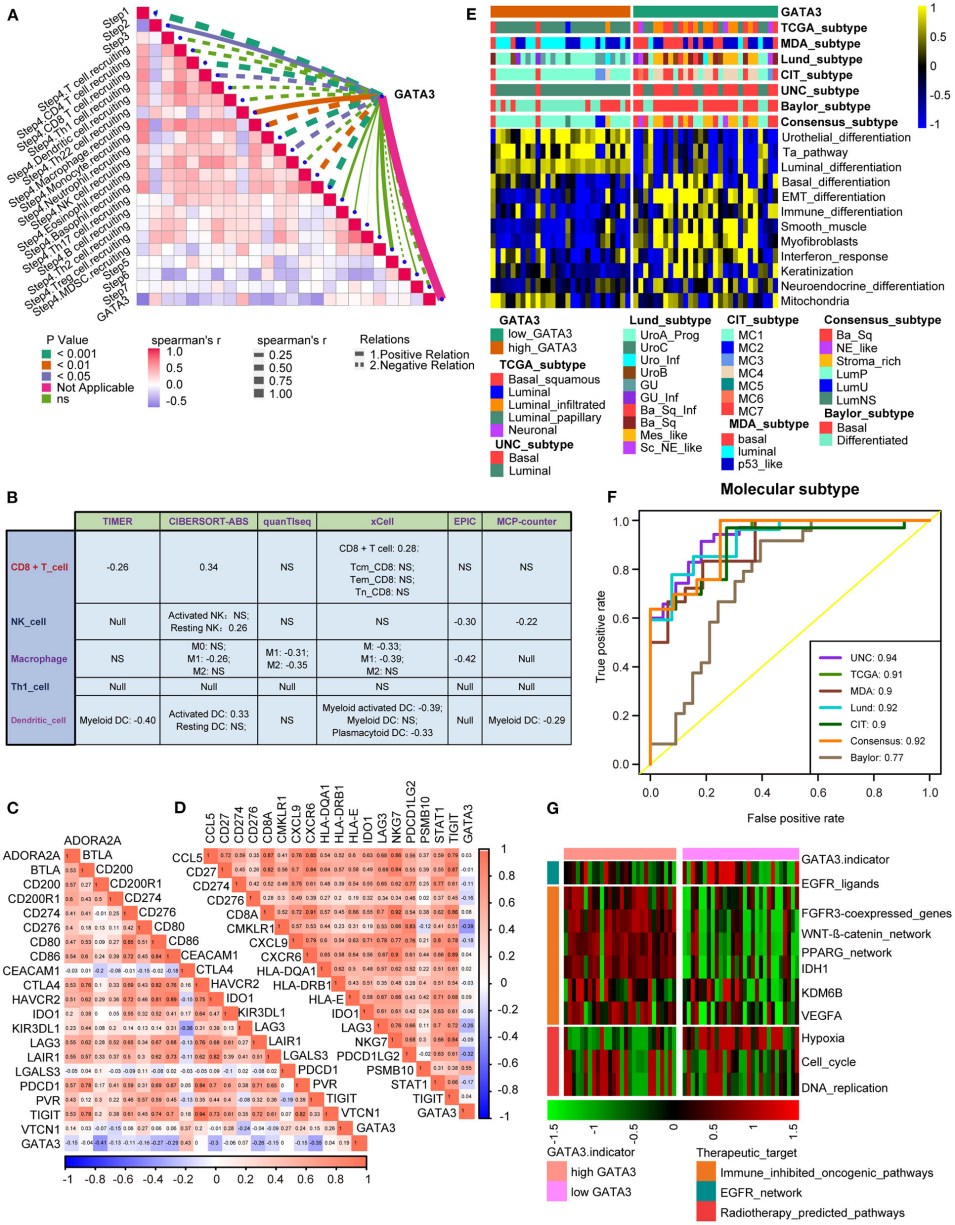
Validation of the GATA3 prediction for molecular subtypes and the response to several therapies in the Xiangya-Pingkuang cohort. **(A)** Correlations between GATA3 and the process of the anti-cancer immunity cycle. **(B)** Correlations between GATA3 and the infiltration levels of five tumor-associated immune cells. **(C)** Correlations between GATA3 and 20 immune checkpoints. **(D)** Correlation between GATA3 and TIS-related effector genes. **(E)** Correlations between GATA3 and seven molecular subtype systems. **(F)** GATA3 molecular subtype prediction accuracy. **(G)** Correlation between GATA3 and the enrichment scores of therapeutic signatures.

In the Xiangya-Pingkuang cohort, GATA3 was validated to be negatively correlated with the majority of the cancer-immune cycles, especially with their vital steps, such as T cell recruiting, Macrophage recruiting, and NK cell recruiting (Figure 5A). We also performed the correlation analysis between GATA3 and CD8+ T-cells, NK cells, dendritic cells, Th1 cells, and macrophages in multiple algorithms. And GATA3 was negatively associated with those immune cells (Figure 5B). Subsequently, GATA3 was found to be negatively correlated with immune checkpoints, including CD274, LAG-3, and CTLA-4 (Figure 5C). In addition, the negative correlation between GATA3 with TIS (T-cell inflamed scores) effector genes including CD274, LAG-3, and TIGHT was confirmed (Figure 5D). Furthermore, the finding that BLCA with low GATA3 was more likely to be the basal subtype was validated in our cohort (Figure 5E). Consistently, high GATA3 indicated luminal subtype. Similarly, AUC in predicting molecular subtypes was ≥0.90 except for the Baylor system (Figure 5F). Likewise, low GATA3 showed poorer response to immune inhibited oncogenic therapy (Figure 5G). Results in the Xiangya-Pingkuang cohort were consistent with those from the TCGA-BLCA cohort.

### GATA3 Predicted Clinical Response of ICB in the IMvigor210 Cohort

**Figure 6:**
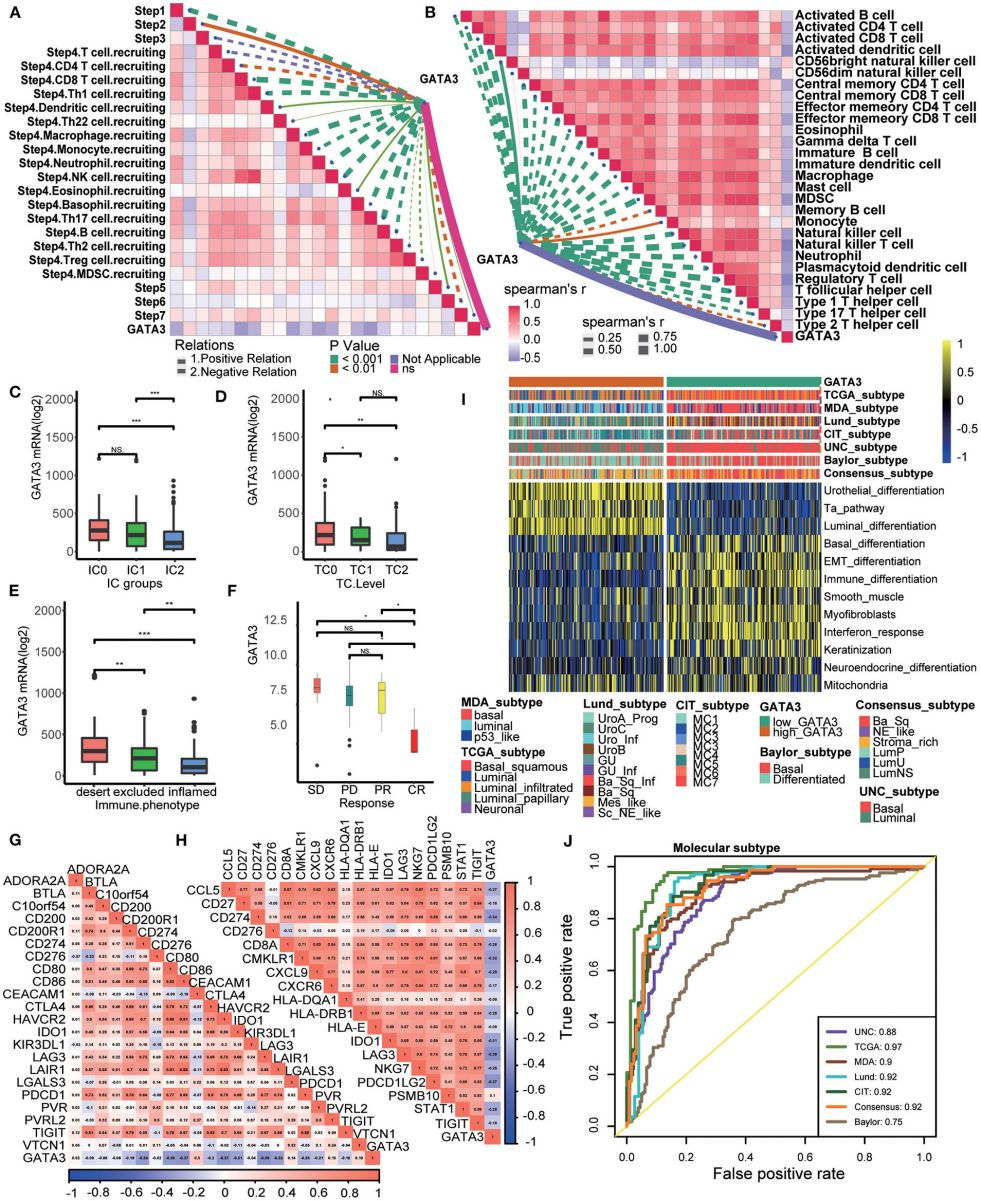
Validation of the GATA3 prediction for low immune infiltration and molecular subtypes in the IMvigor210. **(A)** Correlation between GATA3 and the process of the anti-cancer immunity cycle. **(B)** Correlation between GATA3 and several immune-related cells. **(C)** Differential expression of GATA3 in different IC groups. **(D)** Differential expression of GATA3 in different TC groups. **(E)** Differential expression of GATA3 in three immune phenotypes (the desert, excluded, and inflamed). **(F)** Correlation between GATA3 and the clinical response of tumor immunotherapy in the desert group. **(G)** Correlation between GATA3 and ICI effector genes. **(H)** Correlation between GATA3 and TIS-related genes. **(I,J)** Correlations between GATA3 and seven molecular subtype systems and corresponding ROC analysis for prediction accuracy.

Patients in the IMvigor210 cohort received anti-PD-1 therapy. In this cohort, we comprehensively correlated GATA3 with the TME immune status and the clinical response of ICB. Results showed significantly negative correlations between GATA3 and the activities of several anticancer immunity cycles (Figure 6A). As a result, the infiltration level of TIICs was also negatively related to GATA3 expression (Figure 6B). The PD-L1 expression on immune cells or cancer cells was detected using immunohistochemistry(IHC) in the IMvogor210 cohort. Then, patients were classified into several groups based on the PD-L1 expression. Here, we found that GATA3 was highly expressed in TC0 (tumor cells with the lowest PD-L1 expression) group and IC0 (immune cells with the lowest PD-L1 expression) group compared to other groups (Figures 6C,D). Besides, the expression of GATA3 was the highest in the deserted phenotypes as well (Figure 6E). Moreover, GATA3 was negatively correlated with the expression of several ICI genes and the TIS genes (Figures 6G,H). All results validated that GATA3 shaped a cold TME with a low immune infiltration level. As expected, GATA3 was significantly down-regulated in patients with complete response to ICB (Figure 6F). Finally, the associations between GATA3 and molecular subtypes in the IMvigor210 cohort were consistent with those in the TCGA-BLCA and the Xiangya-Pingkuang cohorts. Low GATA3 expression indicated basal subtype, and the AUCs of GATA3 in predicting subtypes were acceptable (Figures 6I,J).

## Discussion

GATA3, standing for GATA binding protein 3, contains two GATA-type zinc fingers at the structural level and is closely associated with the progression of various cancers, including breast cancer (8, 32–34). GATA3 can also be considered a sensitive and specific marker for urothelial carcinoma (35) and as a criterion for BLCA subtypes classification. Patients with basal/squamous (BASQ)-like tumors, namely those with low GATA3 expression, were more likely to achieve a pathological response to platinum-based neoadjuvant chemotherapy (36). In our study, we explored the role of GATA3 in predicting the molecular subtypes in several independent molecular subtypes systems. Notably, GATA3 was a robust molecular subtypes indicator regardless of the different subtype systems.

Although GATA3 can be used as a diagnostic marker for bladder cancer, many studies demonstrated that GATA3 inhibited cancer cell migration, invasion, and EMT. Thus, the loss of GATA3 was consistently observed in high-grade invasive bladder cancer (6). Similarly, we also observed a similar phenomenon; we found that GATA3 over-expression indicated favorable prognosis, lower tumor grade, and lower tumor stage. Besides, we found that GATA3 was also highly expressed in papillary tumors, which to some extent explained the fact that the prognosis of papillary tumors was better than that of non- papillary tumors. In addition, we found consistent results in the Xiangya-Pingkuang cohort, which further demonstrated the robustness of our analyses.

GATA3 can master vital biological processes, including T-cell development, proliferation, and maintenance in immune regulation (37). In particular, GATA3 was considered a critical transcription factor in the differentiation of T helper 2 (Th2) and was found to play a crucial part in innate hematopoietic and lymphoid-cell development (15). Besides, Notch signals affected GATA3 directly in Th2 cell development, which indicated Notch signaling and GATA3 intersecting (15, 38–40). In addition, GATA3 inhibited IFN-γ through interacting with Runt-related transcription factor 3 (Runx3) (41). These studies supported that GATA3 may play a critical role in modulating the anti-cancer immunity of TME.

Our work reconfirmed the previous deduction in that we found that GATA3 was negatively correlated with a majority of immunomodulators which were necessary for the recruitment of immune cells in the TME. Besides, we also confirmed that GATA3 was negatively correlated with the infiltration level of CD8 T cells, CD4 T-cells, and dendritic cells, indicating that tumors with high GATA3 were more likely to be non-inflamed subtypes, which would be insensitive to immune-checkpoint blocking therapy (16). GATA3 was also negatively correlated with immune checkpoints, including PD-L1, PD-1, CTLA-4, and LAG-3. The expression of these molecules was also positively related to the efficacy of immunotherapy (42). More importantly, we verified directly in the IMvigor210 cohort that GATA3 was closely associated with the effectiveness of ICB. Overall, low GATA3 indicated a basal subtype, with better response to ICB and neoadjuvant therapy, while high GATA3 indicated a luminal subtype, which was more responsive to targeted therapy, such as the blocking GATA3, β-catenin, PPAR-γ, and FGFR3 pathways, and anti-angiogenic therapy.

Inevitably, flaws existed in several aspects of our study. First, all of the results, including the molecular and pan-cancer analyses, were based on bioinformatic analyses without *in vivo* or *in vitro* experiments. Second, we validated the results in the Xiangya-Pingkuang cohort with 57 BLCA samples only, which was not sufficient enough for a pronounced verification. Thus, more data from BLCA tumor tissues and further experiments are urgently needed.

## Conclusion

In conclusion, results suggested that high GATA3 expression promoted BLCA to form a non-inflammatory immune TME resistant to cancer immunotherapy. Therefore, blocking GATA3 may enhance the sensitivity of immunotherapy. Meanwhile, GATA3 can also be used as a response predictor of several treatment options, such as immunotherapy, chemotherapy, radiotherapy, and targeted therapy.

## Data Availability Statement

The original contributions presented in the study are included in the article/Supplementary Materials, further inquiries can be directed to the corresponding author/s.

## Author Contributions

QZ and PL: conception and design and collection and assembly of data. BQ, TQ, XL, and YY: provision of study materials or patients. QZ, PL, YL, and QW: data analysis and interpretation. All authors contributed to the article, manuscript writing, and approved the submitted version.

## Funding

This work was funded by The Youth Science Foundation of Xiangya Hospital, Central South University (2019Q09), Natural Science Foundation of Changsha (kq2014274), Natural Science Foundation of Hunan Province (2021jj31082), and National Natural Science Foundation of China (82103298).

## Conflict of Interest

The authors declare that the research was conducted in the absence of any commercial or financial relationships that could be construed as a potential conflict of interest. The handling Editor (QZ) declared a shared affiliation with the authors (PL, TQ, XL, YY, and BQ).

## Publisher's Note

All claims expressed in this article are solely those of the authors and do not necessarily represent those of their affiliated organizations, or those of the publisher, the editors and the reviewers. Any product that may be evaluated in this article, or claim that may be made by its manufacturer, is not guaranteed or endorsed by the publisher.

## Supplementary Material

Supplementary Figure 1 **(A)** The workflow of this study. **(B)** Single-cell analysis of GATA3 expression in tumor and normal tissues. **(C)** Correlations between GATA3 and molecular subtypes with seven different subtyping systems in GSE32894. **(D)** ROC analysis on the prediction accuracy of GATA3 for molecular subtypes with different systems in GSE32894. **(E,F)** The same analytical methods as those in Supplementary Figures 1C,D used in GSE31684.

## Abbreviations

BLCA: bladder cancer
TCGA: The Cancer Genome Atlas
TME: tumor microenvironment
TIICs: tumor-infiltrating immune cells
TIS: T-cell inflamed scores
HR: hazard ratios
OS: overall survival
TILs: tumor-infiltrating lymphocytes
ICIs: checkpoint inhibitors
ROC: receiver operating characteristic
AUC: area under curve
ICB: immune checkpoint blockade
IHC: immunohistochemistry
TC0: tumor cells with the lowest PD-L1 expression
IC0: immune cells with the lowest PD-L1 expression
BASQ: basal/squamous
Th2: T helper 2
Runx3: RUNX Family transcription factor 3.

## References

[1] Bray F Ferlay J Soerjomataram I Siegel RL Torre LA Jemal A. Global cancer statistics 2018: GLOBOCAN estimates of incidence and mortality worldwide for 36 cancers in 185 countries. CA Cancer J Clin. (2018) 68:394–424. 10.3322/caac.21492 30207593

[2] Lenis AT Lec PM Chamie K Mshs MD. Bladder Cancer: a review. Jama. (2020) 324:1980–91. 10.1001/jama.2020.17598 33201207

[3] Lentjes MH Niessen HE Akiyama Y de Bruïne AP Melotte V van Engeland M. The emerging role of GATA transcription factors in development and disease. Expert Rev Mol Med. (2016) 18:e3. 10.1017/erm.2016.2 26953528 PMC4836206

[4] Fatima N Osunkoya AO. GATA3 expression in sarcomatoid urothelial carcinoma of the bladder. Hum Pathol. (2014) 45:1625–9. 10.1016/j.humpath.2014.03.015 24824028

[5] Higgins JP Kaygusuz G Wang L Montgomery K Mason V Zhu SX. Placental S100 (S100P) and GATA3: markers for transitional epithelium and urothelial carcinoma discovered by complementary DNA microarray. Am J Surg Pathol. (2007) 31:673–80. 10.1097/01.pas.0000213438.01278.5f 17460449

[6] Li Y Ishiguro H Kawahara T Kashiwagi E Izumi K Miyamoto H. Loss of GATA3 in bladder cancer promotes cell migration and invasion. Cancer Biol Ther. (2014) 15:428–35. 10.4161/cbt.27631 24448324 PMC3979820

[7] Bejrananda T Kanjanapradit K Saetang J Sangkhathat S. Impact of immunohistochemistry-based subtyping of GATA3, CK20, CK5/6, and CK14 expression on survival after radical cystectomy for muscle-invasive bladder cancer. Sci Rep. (2021) 11:21186. 10.1038/s41598-021-00628-5 34707176 PMC8551252

[8] Bernardo C Monteiro FL Direito I Amado F Afreixo V Santos LL. Association between estrogen receptors and GATA3 in bladder cancer: a systematic review and meta-analysis of their clinicopathological significance. Front Endocrinol (Lausanne). (2021) 12:684140. 10.3389/fendo.2021.684140 34690921 PMC8531553

[9] Barth I Schneider U Grimm T Karl A Horst D Gaisa NT. Progression of urothelial carcinoma in situ of the urinary bladder: a switch from luminal to basal phenotype and related therapeutic implications. Virchows Arch. (2018) 472:749–58. 10.1007/s00428-018-2354-9 29654370 PMC5978840

[10] Warrick JI Walter V Yamashita H Chung E Shuman L Amponsa VO. FOXA1, GATA3 and PPAR? cooperate to drive luminal subtype in bladder cancer: a molecular analysis of established human cell lines. Sci Rep. (2016) 6:38531. 10.1038/srep38531 27924948 PMC5141480

[11] Choi W Porten S Kim S Willis D Plimack ER Hoffman-Censits J. Identification of distinct basal and luminal subtypes of muscle-invasive bladder cancer with different sensitivities to frontline chemotherapy. Cancer Cell. (2014) 25:152–65. 10.1016/j.ccr.2014.01.009 24525232 PMC4011497

[12] Kamoun A de Reyniès A Allory Y Sjödahl G Robertson AG Seiler R. Aconsensus molecular classification of muscle-invasive bladder cancer. Eur Urol. (2020) 77:420–33. 10.1016/j.eururo.2019.09.006 31563503 PMC7690647

[13] Yu F Sharma S Edwards J Feigenbaum L Zhu J. Dynamic expression of transcription factors T-bet and GATA-3 by regulatory T-cells maintains immunotolerance. Nat Immunol. (2015) 16:197–206. 10.1038/ni.3053 25501630 PMC4297509

[14] Tindemans I Serafini N Di Santo JP Hendriks RW. GATA-3 function in innate and adaptive immunity. Immunity. (2014) 41:191–206. 10.1016/j.immuni.2014.06.006 25148023

[15] Ho IC Tai TS Pai SY. GATA3 and the T-cell lineage: essential functions before and after T-helper-2-cell differentiation. Nat Revs Immunol. (2009) 9:125–35. 10.1038/nri2476 19151747 PMC2998182

[16] Hu J Yu A Othmane B Qiu D Li H Li C. Siglec15 shapes a non-inflamed tumor microenvironment and predicts the molecular subtype in bladder cancer. Theranostics. (2021) 11:3089–108. 10.7150/thno.53649 33537076 PMC7847675

[17] Liu Z Tang Q Qi T Othmane B Yang Z Chen J. A robust hypoxia risk score predicts the clinical outcomes and tumor microenvironment immune characters in bladder cancer. Front Immunol. (2021) 12:725223. 10.3389/fimmu.2021.791924 34484235 PMC8415032

[18] Chen DS Mellman I. Oncology meets immunology: the cancer-immunity cycle. Immunity. (2013) 39:1–10. 10.1016/j.immuni.2013.07.012 23890059

[19] Ayers M Lunceford J Nebozhyn M Murphy E Loboda A Kaufman DR. IFN-γ-related mRNA profile predicts clinical response to PD-1 blockade. J Clin Invest. (2017) 127:2930–40. 10.1172/JCI91190 28650338 PMC5531419

[20] Robertson AG Kim J Al-Ahmadie H Bellmunt J Guo G Cherniack AD. Comprehensive molecular characterization of muscle-invasive bladder cancer. Cell. (2017) 171: 540–56.e25. 10.1016/j.cell.2017.09.007 PMC5687509 28988769

[21] Rebouissou S Bernard-Pierrot I de Reyniès A Lepage ML Krucker C Chapeaublanc E. EGFR as a potential therapeutic target for a subset of muscle-invasive bladder cancers presenting a basal-like phenotype. Sci Transl Med. (2014) 6:244ra91. 10.1126/scitranslmed.3008970 25009231

[22] Sjödahl G Lauss M Lövgren K Chebil G Gudjonsson S Veerla S. A molecular taxonomy for urothelial carcinoma. Clin Cancer Res. (2012) 18:3377–86. 10.1158/1078-0432.CCR-12-0077-T 22553347

[23] Damrauer JS Hoadley KA Chism DD Fan C Tiganelli CJ Wobker SE. Intrinsic subtypes of high-grade bladder cancer reflect the hallmarks of breast cancer biology. Proc Natl Acad Sci U S A. (2014) 111:3110–5. 10.1073/pnas.1318376111 24520177 PMC3939870

[24] Mo Q Nikolos F Chen F Tramel Z Lee YC Hayashi K. Prognostic power of a tumor differentiation gene signature for bladder urothelial carcinomas. J Natl Cancer Inst. (2018) 110:448–59. 10.1093/jnci/djx243 29342309 PMC6279371

[25] Wirtz RM Fritz V Stöhr R Hartmann A. [Molecular classification of bladder cancer. Possible similarities to breast cancer]. Pathologe. (2016) 37:52–60. 10.1007/s00292-015-0134-8 26780243

[26] Xu W Xia H Liu W Zheng W Hua L. Exploration of genetics commonness between bladder cancer and breast cancer based on a silcio analysis on disease subtypes. Technol Health Care. (2018) 26:361–77. 10.3233/THC-174699 29758961 PMC6027900

[27] Tokunaga R Zhang W Naseem M Puccini A Berger MD Soni S. CXCL9, CXCL10, CXCL11/CXCR3 axis for immune activation - A target for novel cancer therapy. Cancer Treat Rev. (2018) 63:40–7. 10.1016/j.ctrv.2017.11.007 29207310 PMC5801162

[28] Wu H Zhang X Han D Cao J Tian J. Tumour-associated macrophages mediate the invasion and metastasis of bladder cancer cells through CXCL8. PeerJ. (2020) 8:e8721. 10.7717/peerj.8721 32201645 PMC7073239

[29] Guo CC Bondaruk J Yao H Wang Z Zhang L Lee S. Assessment of luminal and basal phenotypes in bladder cancer. Sci Rep. (2020) 10:9743. 10.1038/s41598-020-66747-7 32546765 PMC7298008

[30] Necchi A Raggi D Gallina A Ross JS Farè E Giannatempo P. Impact of molecular subtyping and immune infiltration on pathological response and outcome following neoadjuvant pembrolizumab in muscle-invasive bladder cancer. Eur Urol. (2020) 77:701–10. 10.1016/j.eururo.2020.02.028 32165065

[31] McConkey DJ Choi W Shen Y Lee IL Porten S Matin SF. A prognostic gene expression signature in the molecular classification of chemotherapy-naïve urothelial cancer is predictive of clinical outcomes from neoadjuvant chemotherapy: a phase 2 trial of dose-dense methotrexate, vinblastine, doxorubicin, and cisplatin with bevacizumab in urothelial cancer. Eur Urol. (2016) 69:855–62. 10.1016/j.eururo.2015.08.034 26343003 PMC4775435

[32] Bertucci F Ng CKY Patsouris A Droin N Piscuoglio S Carbuccia N. Genomic characterization of metastatic breast cancers. Nature. (2019) 569:560–4. 10.1038/s41586-019-1056-z 31118521

[33] Takaku M Grimm SA Wade PA. GATA3 in breast cancer: tumor suppressor or oncogene? Gene Expr. (2015) 16:163–8. 10.3727/105221615X14399878166113 26637396 PMC4758516

[34] Wang W Wang M Xu J Long F Zhan X. Overexpressed GATA3 enhances the sensitivity of colorectal cancer cells to oxaliplatin through regulating MiR-29b. Cancer Cell Int. (2020) 20:339. 10.1186/s12935-020-01424-3 32760217 PMC7379773

[35] Agarwal H Babu S Rana C Kumar M Singhai A Shankhwar SN. Diagnostic utility of GATA3 immunohistochemical expression in urothelial carcinoma. Indian J Pathol Microbiol. (2019) 62:244–50. 10.4103/IJPM.IJPM_228_18 30971548

[36] Font A Domènech M Benítez R Rava M Marqués M Ramírez JL. Immunohistochemistry-based taxonomical classification of bladder cancer predicts response to neoadjuvant chemotherapy. Cancers. (2020) 12.1784 10.3390/cancers12071784 PMC7408104 32635360

[37] Wan YY. GATA3: a master of many trades in immune regulation. Trends Immunol. (2014) 35:233–42. 10.1016/j.it.2014.04.002 24786134 PMC4045638

[38] Ma Y Li J Wang H Chiu Y Kingsley CV Fry D. Combination of PD-1 inhibitor and OX40 agonist induces tumor rejection and immune memory in mouse models of pancreatic cancer. Gastroenterology. (2020) 159:306–19.e12. 10.1053/j.gastro.2020.03.018 32179091 PMC7387152

[39] Fang TC Yashiro-Ohtani Y Del Bianco C Knoblock DM Blacklow SC Pear WS. Notch directly regulates GATA3 expression during T helper 2 cell differentiation. Immunity. (2007) 27:100–10. 10.1016/j.immuni.2007.04.018 17658278 PMC2801546

[40] Tindemans I van Schoonhoven A KleinJan A de Bruijn MJ Lukkes M van Nimwegen M. Notch signaling licenses allergic airway inflammation by promoting Th2 cell lymph node egress. J Clin Invest. (2020) 130:3576–91. 10.1172/JCI128310 32255764 PMC7324208

[41] Yagi R Junttila IS Wei G Urban JF Jr Zhao K Paul WE. The transcription factor GATA3 actively represses RUNX3 protein-regulated production of interferon-gamma. Immunity. (2010) 32:507–17. 10.1016/j.immuni.2010.04.004 20399120 PMC2867662

[42] Nishino M Ramaiya NH Hatabu H Hodi FS. Monitoring immune-checkpoint blockade: response evaluation and biomarker development. Nat Rev Clin Oncol. (2017) 14:655–68. 10.1038/nrclinonc.2017.88 28653677 PMC5650537

