# Supplementary figures and images for: GATA3 Predicts the Tumor Microenvironment Phenotypes and Molecular Subtypes for Bladder Carcinoma
Source: Front Surg. 2022 May 12;9:860663. doi: 10.3389/fsurg.2022.860663 (PMC9135132; doi:10.3389/fsurg.2022.860663)

**A**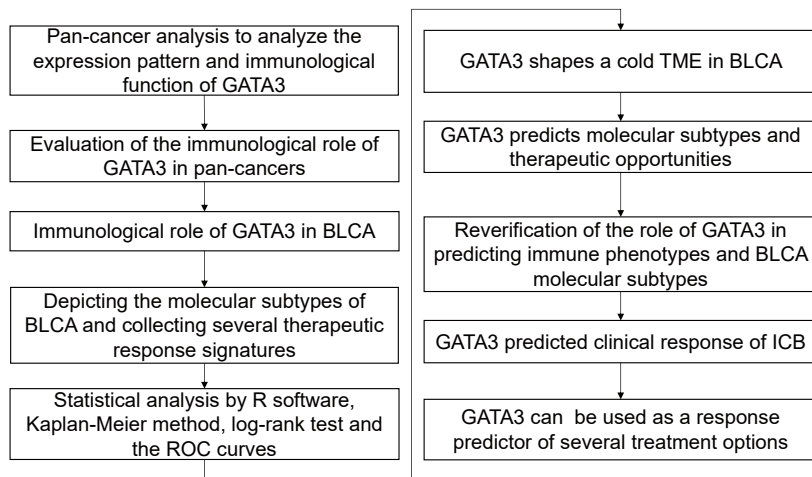**B**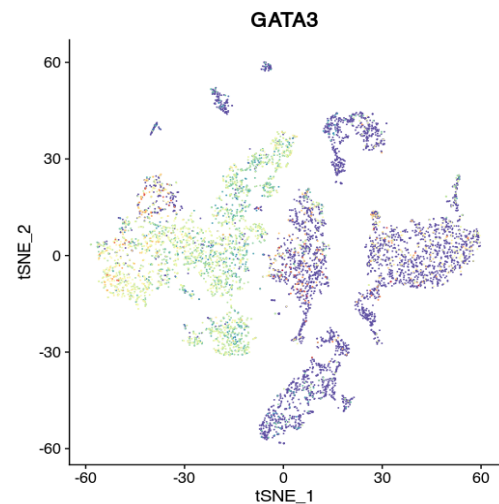**C****GSE32894**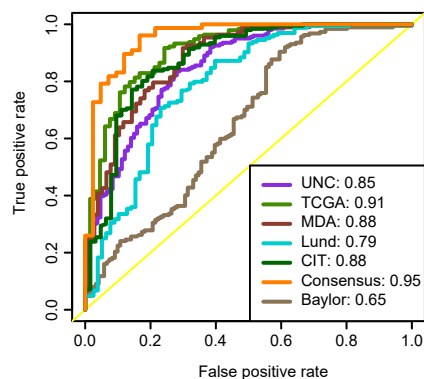**D**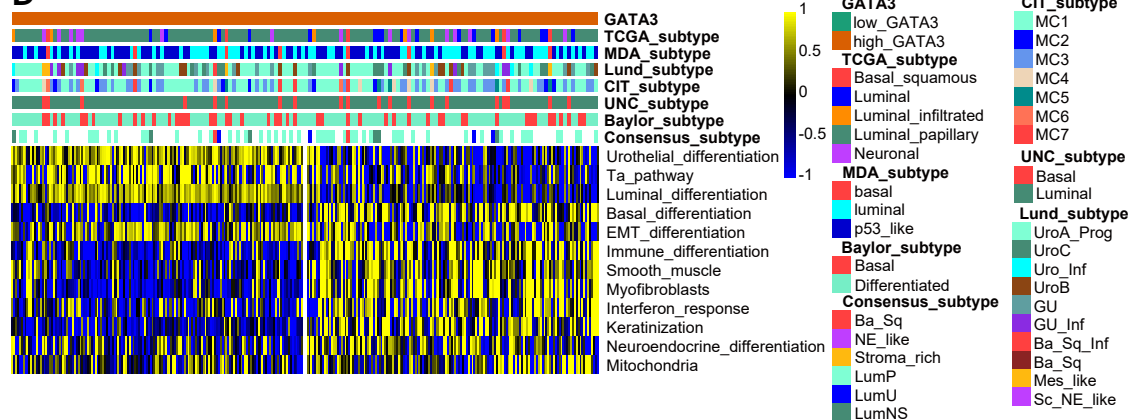**E****Molecular subtype**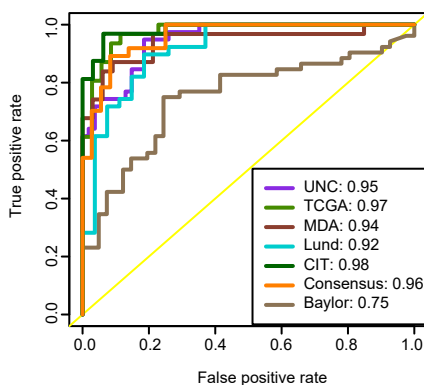**F**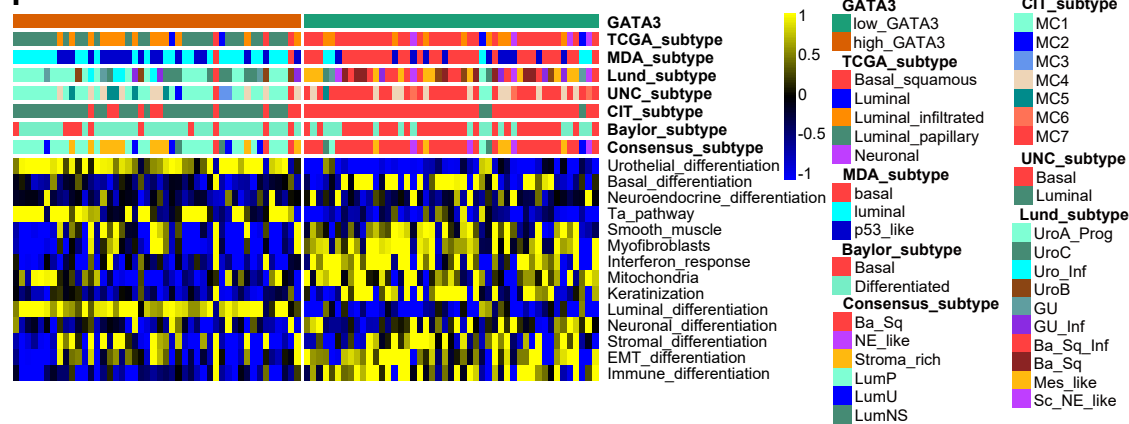

Supplement: Supplementary Figure 1 — (A) The workflow of this study. (B) Single-cell analysis of GATA3 expression in tumor and normal tissues. (C) Correlations between GATA3 and molecular subtypes with seven different subtyping systems in GSE32894. (D) ROC analysis on the prediction accuracy of GATA3 for molecular subtypes with different systems in GSE32894. (E,F) The same analytical methods as those in Supplementary Figures 1C,D used in GSE31684. [file Data_Sheet_1.PDF]
